# FUTURE CARE PLANNING INTERVENTION WITH RURAL OLDER ADULTS

**DOI:** 10.1093/geroni/igz038.1587

**Published:** 2019-11-08

**Authors:** Jeong Eun Lee, Dahee L Kim, Louise Peitz, Eva Kahana, Boaz Kahana

**Affiliations:** 1 Iowa State University, Ames, Iowa, United States; 2 Case Western Reserve University, Cleveland, Ohio, United States; 3 Cleveland State University, Cleveland, Ohio, United States

## Abstract

To address their needs for proactive self-care and end of life planning, we implemented a community based educational program that promotes future care planning (FCP) for community residing older adults. Extension specialists from Iowa State University implemented two brief FCP program sessions with older adults. Topics included both short-term and long-term future care planning activities and strategies. Baseline and post program surveys were completed by 216 community dwelling older adults (M=78.21). The program was successful in getting older Iowans in the rural area start end of life care preparation as well as helping them to make changes in their health care decisions. The majority of participants (89.6 %) reported high satisfaction with the programs. We also found a high rate of change (62%) in opinion regarding future care. The implication of future care planning for is discussed with recommendations for future research.

